# Is less always more? Ceftazidime/avibactam alone or in combination to treat infections caused by *Pseudomonas aeruginosa*: a retrospective observational multi-centre cohort study

**DOI:** 10.1007/s10096-026-05599-x

**Published:** 2026-07-14

**Authors:** Pietro Valsecchi, Marta Colaneri, Andrea Lombardi, Simona Biscarini, Cecilia Azzarà, Alessandra Bandera, Nicolò Corti, Paolo Bonfanti, Leonardo Francesco Rezzonico, Giovanna Travi, Silvia Pontiggia, Stefania Piconi, Arianna Lippi, Mario Tumbarello, Matteo Giovanni Lupi, Angelo Pan, Giacomo Casalini, Spinello Antinori, Andrea Gori, Alessandra Calabresi, Guido Chichino, Virginia Valeria Ferretti, Giulia Gambini, Catherine Klersy, Raffaele Bruno

**Affiliations:** 1 Struttura Complessa Malattie Infettive I, Fondazione I.R.C.C.S. Policlinico San Matteo, Pavia, Italy; 2 Dipartimento di Scienze Biomediche e Cliniche, Università degli Studi di Milano, Milan, Italy; 3 Infectious Diseases Unit, Fondazione IRCCS Ca’ Granda Ospedale Maggiore Policlinico, Milan, Italy; 4 Department of Pathophysiology and Transplantation, University of Milan, Milan, Italy; 5 University of Milano-Bicocca, Fondazione IRCCS San Gerardo dei Tintori, Milan, Italy; 6 Division of Infectious Diseases, ASST Grande Ospedale Metropolitano Niguarda, Milan, Italy; 7 Dipartimento di Scienze Clinico-chirurgiche, Diagnostiche e Pediatriche, Università degli Studi di Pavia, Pavia, Italy; 8 Infectious Diseases Unit, A. Manzoni Hospital, Via dell’ Eremo 9, Lecco, Italy; 9 Infectious and Tropical Diseases Unit, Department of Medical Sciences, University Hospital of Siena, Siena, Italy; 10 Department of Medical Biotechnologies, University of Siena, Siena, Italy; 11 Infectious Disease Unit, ASST Cremona, Cremona, Italy; 12 III Division of Infectious Diseases, Luigi Sacco Hospital, ASST Fatebenefratelli Sacco, Milan, Italy; 13 SOC Malattie Infettive, AOU SS Antonio e Biagio e C. Arrigo, Alessandria, Italy; 14 SSD Biostatistica e Clinical Trial Center, Fondazione I.R.C.C.S. Policlinico San Matteo, Pavia, Italy

**Keywords:** *Pseudomonas aeruginosa*, Ceftazidime/avibactam, Combination therapy

## Abstract

**Purpose:**

In vitro and in vivo studies suggest adding a second agent to ceftazidime/avibactam (CZA) to enhance bactericidal activity against *Pseudomonas aeruginosa* (PA). Nevertheless, evidence supporting this practice is scarce.

**Methods:**

We conducted a retrospective cohort study of patients admitted to 9 Italian hospitals with microbiologically confirmed PA infection and treated with CZA alone or in combination from 01/01/2015 to 31/12/2021. The primary endpoint was 14-day clinical cure. Secondary endpoints were 28-day all-cause mortality, 56-day microbiological failure, 56-day infection relapse, and emergence of CZA resistance. A generalized linear model extended to the binomial family, with identity link, weighted for IPTW (inverse probability of treatment weighting), was fitted to compare the two groups for 14-day clinical cure. Results were reported in terms of risk difference (RD) and 90% Confidence Interval (90%CI).

**Results:**

Eighty-seven patients with infection due to PA were included; 17 were treated with CZA monotherapy, and 70 with combination therapy with CZA plus another anti-pseudomonal agent. Sequential organ failure assessment was significantly higher (median 6 [3–9]) in the combination group compared to monotherapy (median 3 [1–5]; *p* = 0.049). After weighting patients according to the IPTW, CZA monotherapy did not reach the clinical equipoise to combination therapy in terms of 14-day clinical cure (RD -0.28, 90% CI -0.41 to -0.14). No significant risk difference between groups was found for any of the secondary endpoints.

**Conclusion:**

Our findings suggest that combination therapy with CZA for treating PA infections may provide some clinical benefit over monotherapy despite the absence of a definitive survival advantage.

**Supplementary Information:**

The online version contains supplementary material available at https://doi.org/10.1007/s10096-026-05599-x.

## Background

*Pseudomonas aeruginosa* (PA) is a frequent cause of healthcare-associated infections, with a mortality rate exceeding 40% in severe cases such as bloodstream infections (BSIs) and ventilator-associated pneumonia (VAP) [1, 2]. Antimicrobial resistance is a significant concern, as more than 17.5% of the isolates are resistant to at least two antibiotics [3, 4]. Due to its resistance profile, the World Health Organization included PA among the high-priority pathogens in terms of antimicrobial resistance [5].

Multi-drug resistant (MDR) PA is defined as being non-susceptible to at least one antimicrobial in at least three antimicrobial classes to which it is usually susceptible, while extensively drug-resistant (XDR) isolates are resistant to all but two or fewer classes [6]. In 2018, the concept of difficult-to-treat resistance (DTR) was introduced, describing PA resistant to key-antibiotics such as ceftazidime, cefepime, piperacillin/tazobactam, imipenem-cilastatin, meropenem, ciprofloxacin, levofloxacin, and aztreonam [7].

Ceftazidime/avibactam (CZA) is a β-lactam/β-lactamase inhibitor combination of the third-generation cephalosporin ceftazidime and the β-lactamase inhibitor avibactam. The combination with avibactam restores ceftazidime activity against a broad range of Gram-negative bacteria, including ESBL-, AmpC-, KPC and OXA 48-producing *Enterobacterales* and PA, but not against metallo-β-lactamase (MBL) producers [8]. 

CZA represents a valuable therapeutic option against PA isolates, as susceptibility rates range from 85% to 95% according to several epidemiological studies [9, 10]. Nevertheless, the emergence of clinical isolates resistant to CZA has been increasingly described since the introduction of the drug in 2015 [11, 12]. 

Emerging resistance to CZA in PA is mediated by several mechanisms, including OXA-2 and OXA-10 mutations and modifications at the catalytic site of AmpC enzymes, leading to enhanced ceftazidime hydrolysis and increased resistance to avibactam [13–16]. Other mechanisms of resistance include target site modifications involving penicillin-binding proteins (PBPs), porin mutations (OprD), and efflux pumps [11, 16]. 

One proposed strategy to counteract resistance is the combination of two active antibiotics. However, while this approach has shown promising results in vitro and in vivo animal studies, translation of this data in clinical practice requires further evidence [17, 18]. 

Whether combining a second antipseudomonal agent with CZA would improve patients’ outcomes and reduce the emergence of CZA-resistant PA strains is still unclear [19, 20]. 

Due to insufficient clinical data, the 2022 guidelines from the European Society of Clinical Microbiology and Infectious Diseases do not recommend for or against combination therapy with CZA for carbapenem-resistant PA infections, while the Infectious Diseases Society of America 2023 guidance document advises against routine combination therapy for DTR PA [21, 22]. 

We hypothesize that CZA monotherapy would provide similar clinical and microbiological outcomes compared to CZA combined with other active antimicrobials for infections caused by PA. To test this hypothesis, we retrospectively collected data from multiple centres in Northern Italy and compared outcomes between the two treatment strategies.

## Methods

### Study design and participants

This was an explorative multicentre retrospective cohort study. Data from adult patients with microbiologically confirmed PA infection admitted to the participating centres between the 1st of January 2015 and the 31st of December 2021 and treated with CZA for at least 72 h were retrospectively collected.

Patients were excluded if < 18 years of age, if antibiotic treatment was empirically driven by colonization without an available bacterial isolate and antimicrobial susceptibility testing, if isolates were obtained from cultures of drainage sites after 48 h from drainage placement or swabs from non-sterile sites (e.g. chronic ulcers, wounds or intact skin), or if there was no assessment for the primary endpoint.

### Objectives of the study

The primary endpoint of the study was the 14-day clinical cure, defined as resolution of signs and symptoms of infection. Specific infection types were defined according to CDC/NHSN Surveillance Definitions for Specific Types of Infections [23]. Clinical cure definitions were developed a priori for each infection and are presented in the Supplementary materials.

Secondary endpoints included:

- Microbiologic failure within 56 days, defined as persistent positive cultures for the index pathogen from the same infection site. (endpoint 2.1)
- Infection relapse within 56 days, defined as the recurrence of the same type of PA infection after achieving clinical cure. (endpoint 2.2)
- Emergence of CZA resistance in follow-up isolates, defined as a new isolate with CZA minimum inhibitory concentrations (MIC) above the breakpoint. (endpoint 2.3)
- 28-day all-cause mortality. (endpoint 2.4)

Both the primary and secondary endpoints were calculated from the index culture collection.

### Variables and definitions

Data were retrospectively retrieved from elec tronic health records by a dedicated infectious disease specialist at each participating centre.

For each patient, demographic data (age, gender), Charlson comorbidity index (CCI) [24] and presence of comorbidities, including those related to an increased risk for PA infection (cystic fibrosis, bronchiectasis, chronic obstructive pulmonary disease, active immunosuppression, neutrophil blood count < 500 cells/mm), were recorded [24–26]. Other relevant variables were intensive care unit (ICU) stay, estimated glomerular filtration rate (eGFR), need for renal replacement therapy, and any prior treatment with anti-pseudomonal antibiotics within the past 12 months.

Severity of infection onset was assessed using the Sequential Organ Failure Assessment (SOFA) score. The presence of septic shock was defined according to Sepsis-3 criteria as sepsis with persistent hypotension despite adequate fluid replacement and/or blood lactates > 1,6 mmol/L [27]. Presence of adequate source control was defined as all the acts aimed to remove the putative source of infection or to reduce the burden of infection, such as central line or urinary catheter removal, abscess drainage, and surgical debridement or removal of infected devices.

CZA was administered either by continuous infusion (over the entire dosing interval) or extended infusion (lasting at least 3 h).

### Microbiological methods

Clinical isolates were identified in each centre’s Microbiology Unit from biological samples, and antimicrobial susceptibility testing was performed in line with local standard procedures. Results were interpreted according to EUCAST clinical breakpoints [28]. MDR, XDR, and DTR isolates were defined as described above [6]. Combination therapy was considered effective when it was interpreted as susceptible or dose-dependent, according to EUCAST clinical breakpoints [28]; in the absence of clinical breakpoints for aminoglycosides and fosfomycin, their use in combination was interpreted as conforming to respective EUCAST rationale documents [29, 30].

### Statistical methods

All statistical analyses were performed using Stata 18 (StataCorp. 2023. Stata Statistical Software: Release 18. College Station, TX: StataCorp LLC.). Two-sided type I error was set at 0.1.

Qualitative variables were described as counts and percentages of each category. Quantitative variables have been summarized as median and Interquartile Range (IQR). The association between 2 qualitative variables was evaluated via Fisher’s exact test. Quantitative variables were compared between two groups using the Mann-Whitney test.

Propensity score-based methods were used to compare outcomes between CZA monotherapy and CZA combination therapy for the primary endpoint only, to control for confounding and reduce imbalances between the two treatment groups. Adjustment was not extended to secondary endpoints to preserve statistical power. After trimming the extreme 1% of observations, the data were weighted using the Inverse Probability of Treatment Weighting (IPTW) method.

Data were captured for patients treated with CZA alone versus those treated with CZA in combination. The following covariates were selected for propensity score calculation due to their clinical relevance: age at treatment, CCI, active immunosuppression, treatment with an anti-pseudomonal drug in the last 12 months, ICU admission, hospital-acquired pneumonia (HAP) /VAP, BSI, MDR, XDR, and DTR.

We included MDR, XDR, and DTR in the model since the study focused on targeted treatment, and this information was available to the treating physician when selecting antimicrobial therapy.

Multiple imputation was not performed due to the negligible amount of missing data for these variables.

A generalized linear model extended to the binomial family, with identity link (weighted for IPTW), was fitted to compare CZA alone and CZA combined for 14-day clinical cure (primary endpoint). Results of this model were reported in terms of risk difference (RD) with 90% Confidence Interval (90% CI).

Due to the exploratory nature of the study and the area of uncertainty over the effect of combination therapy on clinical cure, we sought to evaluate clinical equipoise between the two treatment arms. A lower limit of the confidence interval not exceeding − 10% was set to ensure that the two treatment groups were comparable.

The same analysis (without weighting for IPTW) was performed to compare CZA alone and CZA combined for microbiologic failure, infection relapse, and emergence of CZA resistance (secondary endpoints 2.1, 2.2, 2.3).

Kaplan-Meier product limit method and Cox regression model were used to estimate 28-day all-cause mortality and to compare them between groups. Results from Cox models were reported in terms of Hazard Ratio (HR) with 90%CI.

## Results

### Study population

Data from 87 patients treated with CZA for *P. aeruginosa* infection in the participating centres were retrieved, 17 treated with CZA monotherapy and 70 with a combination of CZA and one or more antimicrobials. Detailed demographic and clinical representation of the groups is shown in Table 1.

**Table 1:** Demographic and clinical characteristics of the study population according to treatment group. Data are presented as median [IQR] for continuous measures, and *n* (%) for categorical measures, and *p* values refer to Wilcoxon rank sum test for continuous variables and to Fisher exact test for categorical variables

	CZA Monotherapy	CZA in Combination therapy	*p*
	*N* = 17	*N* = 70	
Age, median (IQR)	72 [62–75]	62 [52–69]	**0.018**
Female, *n* (%)	4 (23.5)	17 (24.3)	>0.9
Charlson comorbidity index median (IQR) Missing = 2	4 [3–5]	3 [2–5]	0.088
DM II, *n* (%) Missing = 3	2 (12.5)	16 (22.9)	0.50
COPD, *n* (%) Missing = 3	2 (11.8)	5 ( 7.2)	0.62
Solid tumor, *n* (%) Missing = 2	1 (5.9)	9 (12.9)	0.68
Hematological malignancy, *n* (%) Missing = 2	4 (23.5)	9 (12.9)	0.27
Active immunosuppression, *n* (%) Missing = 4	3 (17.6)	16 (23.5)	0.75
Cystic fibrosis, *n* (%) Missing = 2	1 (5.9)	7 (10.0)	>0.9
Treatment with anti-pseudomonal drug in the last 12 months, *n* (%) Missing = 6	10 (58.8)	48 (71.6)	0.38
eGFR (ml/min), *n* (%) Missing = 11			**0.048**
=> 120	1 (7.1)	13 (20.0)	
50 < = x < 120	8 (57.1)	40 (61.5)	
30 < = X < 50	4 (28.6)	4 (6.2)	
15 < = X < 30	1 (7.1)	1 (1.5)	
X < 15	0 (0.0)	7 (10.8)	
Renal replacement, *n* (%) Missing = 2	4 (23.5)	22 (31.4)	0.77
CRRT, n (%)	4 (100.0)	21 (95.5)	>0.9
Sequential Organ Failure Assessment (SOFA) Score, median (IQR) Missing = 30	3 [1–5]	6 [3–9]	**0.049**
ICU admission, *n* (%) Missing = 2	8 (47.1)	48 (68.6)	0.16
Septic shock, *n* (%) Missing = 3	2 (11.8)	22 (31.9)	0.13
Bloodstream infection, *n* (%) Missing = 3	8 (47.1)	20 (29.0)	0.16
Source of infection, *n* (%)			0.19
HAP	4 (26.7)	12 (18.5)	
VAP	5 (33.3)	37 (56.9)	
cIAI	0 (0.0)	2 (3.1)	
UTI	1 (6.7)	2 (3.1)	
cUTI	2 (13.3)	1 (1.5)	
ABSSI	1 (6.7)	6 (9.2)	
Blood catheter	2 (13.3)	5 (7.7)	
Length of treatment, median (IQR)	12 [8–15]	14 [10–18]	0.12
Adequate source control, *n* (%)	7 (43.8)	28 (45.9)	>0.9
Renal adjustment for CZA, *n* (%)	7 (46.7)	24 (34.8)	0.39
CZA extended infusion, *n* (%)	8 (53.3)	38 (58.5)	0.78

*DM* Diabetes mellitus, *COPD* chronic obstructive pulmonary disease, *eGFR* estimated glomerular filtration rate, *CRRT* continuous renal replacement treatment, *HAP* healthcare acquired pneumonia, *VAP* ventilator associated pneumonia, *cIAI* complicated intra-abdominal infection, *UTI* urinary tract infection, *cUTI* complicated urinary tract infection, *ABSSI* acute bacterial skin and soft tissue infection, *CZA* ceftazidime/avibactam

While comorbidities were similar between the two groups, patients receiving CZA monotherapy were significantly older compared to those treated with CZA in combination.

With regards to clinical presentation, the median SOFA score was significantly higher in patients receiving combination treatment compared to those receiving CZA monotherapy. Combination treatment was prescribed in 73% of the cases for hospital-acquired pneumonia HAP or VAP.

While patients in the monotherapy group presented more frequently a reduction in creatinine clearance, dose adjustment according to renal function was not significantly different in the two groups. On the other hand, patients in the combination treatment group presented more frequently with an augmented renal clearance. Detailed information regarding the antimicrobials used in combination is presented in the Supplementary Materials.

Table 2 shows the comparison of variables used to create IPTW, before and after weighting. After weighting, there were no significant differences between the two treatment groups using Hotelling’s t test (*p* = 0.999).

**Table 2:** Comparison of characteristics used to create IPTW. Both raw and weighted descriptives are reported

	Unweighted analyses Hotelling t test: *p* < 0.001		Weighted analyses Hotelling t test: *p* = 0.999	
	Monotherapy (*n* = 17)	Combination (*n* = 70)	Monotherapy (*n* = 11)	Combination (*n* = 33)
Age, year (Mean, SD)	66 (17)	59 (15)	61 (20)	61 (11)
CCI, (Mean, SD)	4.2 (2.0)	3.3 (2.1)	3.2 (2.2)	3.9 (2.2)
Active immunosuppression Yes, (%)	18%	24%	0%	20%
Treatment with anti-pseudomonal drug in the last 12 months, (%)	59%	72%	56%	62%
ICU admission, (%)	47%	69%	81%	62%
HAP/VAP, (%)	29%	55%	42%	37%
Bloodstream infection, (%)	47%	29%	46%	44%
MDR, (%)	59%	48%	51%	59%
XDR, (%)	18%	13%	5%	8%
DTR, (%)	12%	19%	17%	12%

*CCI* Charlson comorbidity index, *ICU* Intensive Care Unit, *VAP* ventilator-associated pneumonia, *MDR* multidrug resistant, *XDR* extremely drug resistant, *DTR* difficult to treat

### Microbiological results

Susceptibility results for the tested antimicrobials and proportions of MDR, XDR, and DTR isolates are shown in Table 3. For one single isolate, CZA MIC was not available, but it was included in the analysis since it was susceptible to ceftazidime alone.

**Table 3:** Antimicrobial resistance according to EUCAST clinical breakpoints between treatment groups. Results are expressed as count and percentages. p-values refer to Fisher exact test. MIC= minimum inhibitory concentration, MDR = multidrug resistant, XDR = extremely drug resistant, DTR= difficult to treat. MIC values are expressed as mg/L

	CZA Monotherapy	CZA in Combination therapy	*p*
	*N* = 17	*N* = 70	
Amikacin R, *n* (%)	5 (29.4)	9 (13.0)	0.19
Aztreonam R, *n* (%) Not performed = 51	4 (23.5)	13 (18.8)	0.91
Cefepime R, *n* (%) Not performed = 16	6 (35.3)	36 (52.2)	0.20
Ceftazidime R, *n* (%) Not performed = 7	6 (35.3)	34 (49.3)	0.28
Piperacillin/tazobactam R, *n* (%)	11 (64.7)	45 (65.2)	0.84
Ciprofloxacin R, *n* (%)	8 (50.0)	32 (47.1)	1.00
Colistin R, *n* (%) Not performed = 8	0 (0.0)	1 (1.5)	0.10
Tobramycin R, *n* (%) Not performed = 30	1 (5.9)	10 (14.5)	0.078
Imipenem R, *n* (%) Not performed = 8	8 (47.1)	44 (64.7)	0.35
Meropenem, *n* (%)			0.44
MIC ≤ 2	5 (31.2)	16 (23.2)	
2 < MIC ≤ 8	1 (6.2)	14 (20.3)	
MIC > 8	10 (62.5)	39 (56.5)	
Ceftolozane/tazobactam R, *n* (%) Not performed = 13	1 (5.9)	8 (11.6)	0.20
Ceftazidime/avibactam, MIC, *n* (%)			0.36
0.5	0 (0)	4 (5.7)	
1	3 (18.7)	1 (1.4)	
2	6 (37.5)	2 (2.9)	
4	3 (18.7)	15 (21.4)	
8	4 (25)	24 (34.3)	
MDR, *n* (%)	10 (58.8%)	33 (47.8%)	0.59
XDR, *n* (%)	3 (17.6%)	9 (13.2%)	0.70
DTR, *n* (%)	2 (11.8%)	13 (19.1%)	0.72

### Primary endpoint: 14-day clinical cure

After 14 days, clinical cure was reached in 40/69 (58%) patients treated with the combination of CZA and in 8/17 (47%) patients treated with CZA monotherapy (RD=-0.11, 90%CI: -0.32 to 0.10).

After weighting patients according to the IPTW, CZA monotherapy did not reach the clinical equipoise to combination therapy for 14-day clinical cure (36.2% vs. 63.8%, RD= -0.28, 90%CI: -0.41 to -0.14).

### Secondary outcomes

At univariable analysis, two groups were comparable for 56 days microbiological failure (RD = 0.13, 90%CI: -0.09 to 0.36), 56 days infection relapse (RD= -0.05, 90%CI: -0.15 to 0.06), and occurrence of CZA resistance (RD= -0.02, 90%CI: -0.10 to 0.07), as described in Table 4.

**Table 4:** Risk differences for secondary outcomes according to treatment groups. Outcomes are presented as counts and percentage. 90%CI

Outcome	Monotherapy	Combination	Risk difference	90% CI for risk difference
56 days microbiological failure	7 (43.7%)	21 (30.4%)	0.13	-0.09 0.36
56 days infection relapse	2 (13.3%)	12 (17.07%)	-0.05	-0.15 0.06
CZA resistance	1 (5.9%)	5 (7.5%)	-0.02	-0.10 0.07

**Fig. 1:**
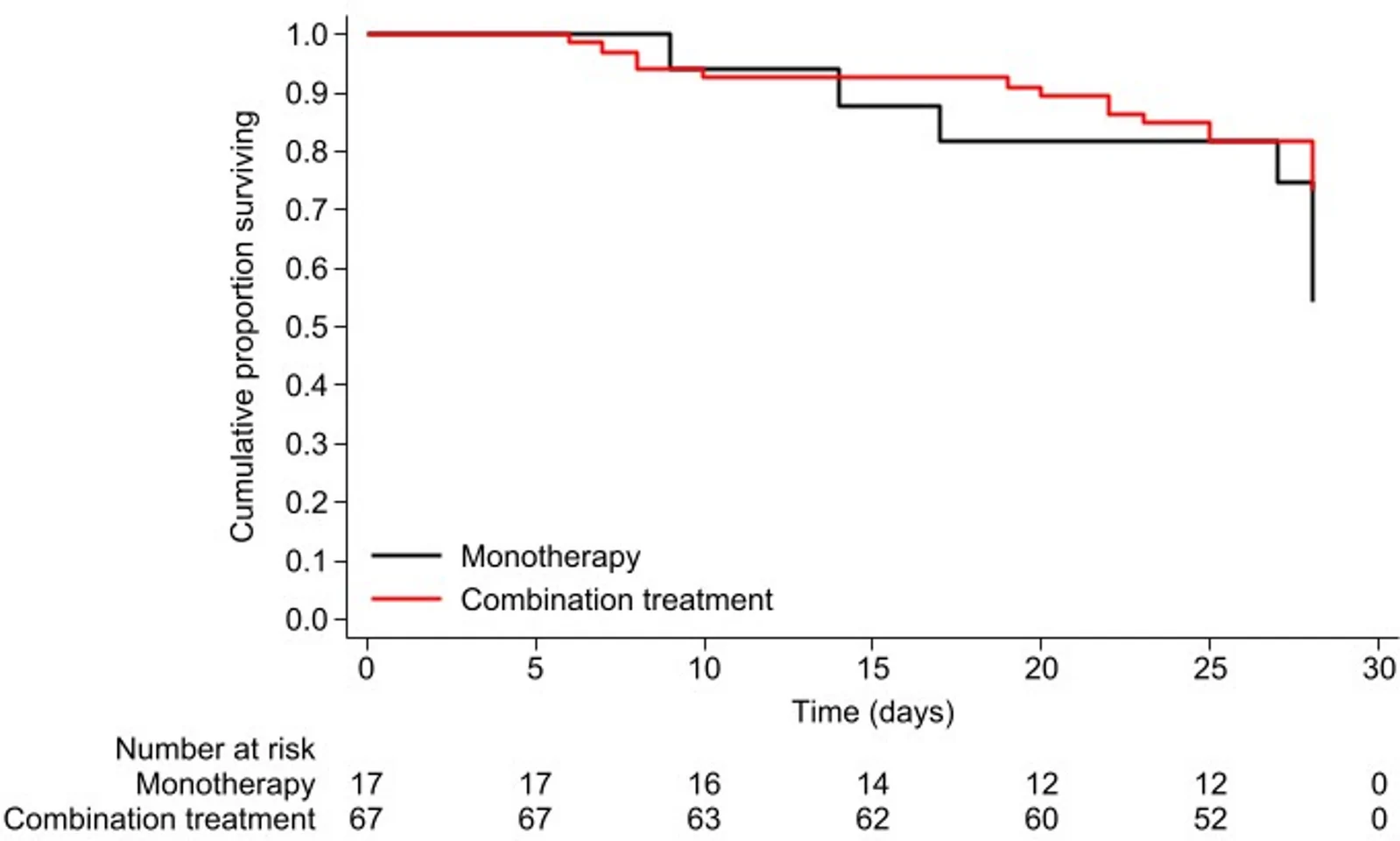
Kaplan-Meier survival curve according to CZA in monotherapy or combination treatment

After 28 days, 24 patients had died: 7 (41.2%) treated with CZA monotherapy and 17 (24.3%) treated with CZA in combination therapy. Again, 28-day all-cause mortality was comparable between groups (Fig. 1, HR = 0.56, 90%CI: 0.23, 1.35).

## Discussion

We found that CZA monotherapy was associated with lower 14-day clinical cure in patients with PA infections when compared to CZA combination with other active antimicrobials. We did not find significant differences between the two regimens for 28-day mortality, 56-day microbiological failure, 56-day infection relapse, and emergence of CZA-resistant isolates. In our cohort, combination therapy was prescribed frequently in patients with pneumonia, higher SOFA scores, and increased renal clearance. On the other hand, patients receiving CZA monotherapy were older and presented with lower renal clearance.

PA represents a significant threat due to the rising incidence of antimicrobial resistance and the associated high mortality in systemic infections [1, 2]. CZA is a key therapeutic option, especially against MDR strains, similar to ceftolozane/tazobactam (C/T) [9]. However, comparative studies between these agents have provided mixed results. While most research shows comparable clinical outcomes, one study reported that C/T resulted in lower recurrence rates and greater cost-effectiveness in patients with PA pneumonia compared to CZA [31–33]. 

Results from a multicentric cohort study by Shields et al. comparing the two agents showed a significantly higher odds of clinical cure in patients treated with C/T compared to CZA for MDR PA infections, and may provide good evidence to support the role of C/T as the first-line agent for these infections [33]. Nevertheless, CZA remains a good option, and it is preferred for isolates resistant to C/T, as it was for 9% of the isolates in this cohort.

Although several studies have demonstrated no significant survival benefit from combination therapy in serious PA infections, including VAP and BSI [34–36], we found that most patients in our cohort—particularly those with lower respiratory tract infections and higher SOFA scores—received combination therapy. Several factors may explain clinicians’ preference for combination therapy in these patients. Although monotherapy is recommended for definitive treatment to avoid toxicity in the absence of clear benefits [21], one retrospective study suggested that empiric combination therapy could be associated with earlier appropriate treatment in patients with VAP [37]. This is important, as inappropriate empiric treatment in severe infections is linked to increased mortality and clinical failure [35]. However, it is worth noting that these results predate the introduction of new β-lactam/β- lactamase inhibitors, which might now reduce the impact of combination therapy on empiric coverage.

Furthermore, β-lactam concentrations in the epithelial lining fluid are lower than in plasma, leading to potential suboptimal antimicrobial concentrations at the infection site, especially in HAP and VAP [38]. Combining two active antibacterial agents can theoretically reduce the risk of clinical failure and selection for antimicrobial resistance due to suboptimal antimicrobial concentrations. This concern is even greater in patients with augmented renal clearance, who are at increased risk of antimicrobial underexposure, as reflected in our cohort, where patients receiving combination treatment had more frequent augmented renal clearance [39].

More than half of the patients in our cohort received CZA as a prolonged or continuous infusion. Prolonged infusion of time-dependent antibiotics such as CZA has been shown to reduce mortality in septic patients [40]. Furthermore, it has been proposed as a strategy to overcome resistance development by targeting aggressive pharmacokinetic/pharmacodynamic (PK/PD) targets, such as a free fraction 100% time above 4 times the MIC [41].

PK/PD optimization is particularly appealing for CZA, which is prone to subtherapeutic exposure due to an unfavourable beta-lactam/beta-lactamase ratio of 1:4 [42]. This strategy has been somewhat alternative to combination therapy, as a study evaluating a pharmacy-driven stewardship intervention to improve target attainment for KPC producing *K. pneumoniae* infection has shown an improvement in clinical and microbiological cure with a reduction in the use of combination therapy [43]. Nevertheless, limited access to therapeutic drug monitoring restricts widespread implementation.

Conversely, patients receiving monotherapy were older and more likely to have reduced renal clearance, reflecting a concern about potential toxicity, particularly nephrotoxicity, in elderly patients. Aminoglycosides and colistin, which were frequently part of the combination regimen, are associated with significant renal toxicity [44, 45].

Interestingly, the proportion of patients with active immunosuppression was similar between the two treatment arms. This subgroup is prone to developing serious PA infections and to being exposed to multiple antibiotic courses [26]; nonetheless, these concerns were not associated with an inclination to choose combination over CZA monotherapy.

We found a notable difference in 14-day clinical cure rates favouring combination therapy. This contrasts with several observational studies comparing monotherapy and combination therapy in serious PA infections, which mainly focused on mortality as the primary outcome [34–36]. Mortality is an objective and highly relevant endpoint, especially given the high mortality rates associated with these infections. In contrast, clinical cure—while more subjective—may reveal smaller differences between treatment groups. To minimize bias, we used rigorous and standardized definitions of clinical cure.

Our findings are at odds with some previous studies on CZA monotherapy versus combination therapy for PA [19, 20]. For instance, Xu et al. reported no significant differences in clinical cure between the two approaches in patients with HAP and VAP [19]. However, the lack of pre-specified time points for evaluating clinical cure makes the comparison difficult. It is possible that combination treatment could offer benefits in the initial phase of treatment, without impacting overall cure rates. Another observational study by Corbella et al. found lower 14-day clinical cure rates with CZA-containing combination regimens, which the authors attributed to residual confounding factors and increased toxicity, although only 3 patients in their cohort experienced antimicrobial-related adverse events [20].

In our cohort, 28-day all-cause mortality was higher than described in the aforementioned studies [19, 20]. This can be explained by the higher proportion of critically ill patients, who accounted for more than half of our sample. Similarly, we did not find a significant difference in mortality between patients treated with CZA monotherapy and patients treated with CZA in combination with other active antimicrobials.

While suppression of further resistance is a theoretical benefit of combination therapy, the proportion of MDR, XDR, and DTR strains at baseline was similar between the two treatment arms. In particular, more than half of the isolates were characterized as MDR, and 14% were XDR. Similarly, 17% of the isolates were characterized as DTR.

The emergence of resistance after treatment with CZA varies from 0% to 40% across studies. Epidemiological differences and differences in resistance definitions could explain this variability [11, 46, 47]. In our cohort, almost 7% of patients developed subsequent resistance, defined as MIC values above the EUCAST clinical breakpoint, without differences between the two groups [28].

Emerging resistance to CZA is mainly mediated by mutations in ampC, combined with the expression of efflux pumps [12, 14, 46–49]. These mutations may lead to increased MIC to other cephalosporins, such as C/T and cefiderocol [50, 51]. A recent comparative study showed lower rates of emerging resistance following treatment with C/T compared to CZA, possibly due to the higher stability of ceftolozane to cephalosporinases [46]. The low rate of resistance in our study can be explained by the smaller number of MDR isolates compared to other cohorts, as several co-resistance mechanisms occur in these isolates and make them more prone to develop further resistance [46, 47, 52].

Our study has some limitations. Firstly, the small sample size limits the generalizability of our findings. Despite including patients from multiple centres with diverse infection types, the overall sample is consistent with previous studies of CZA for PA. Secondly, a shortage of C/T during the study period may have led to increased CZA use, complicating future studies on this topic. Thirdly, the retrospective and observational nature of the study also introduces the possibility of missing data and residual confounding.

Although the presence of missing data was overall limited, the relevant proportion of missing values for the SOFA score precluded the inclusion of an important variable of acute illness severity in the propensity score.

Despite the efforts to reduce confounding through the application of IPTW, we cannot exclude the presence of residual confounding, especially due to the limited sample and unbalanced groups.

The inclusion of several different types of infection could introduce substantial heterogeneity, given the limited sample size. Since the majority of patients presented with HAP/VAP, the results may be driven mainly by this subgroup, although distribution within treatment groups was balanced using propensity score.

Since this study was pathogen-directed, microbiological identification of PA was required to define clinical relapse, thus possibly excluding patients presenting with recurrent signs/symptoms of infection that do not receive adequate microbiological work-up.

Furthermore, the impact of COVID-19 cannot be overlooked, as it complicates the evaluation of clinical cure in patients with lower respiratory tract infections and viral pneumonia. Finally, cystic fibrosis was present in 10% of patients in our cohort, further complicating cure evaluation in these cases.

Our findings suggest that the debate between monotherapy and combination therapy for PA infections, particularly with CZA, remains unresolved despite the lack of a clear survival benefit for combination therapy. Due to the study design and the aforementioned limitations, these results should be considered hypothesis-generating.

While monotherapy offers the benefit of lower toxicity, especially in elderly patients, combination therapy—at least in the early phase—might be associated with higher clinical cure rates by improving empirical coverage or reducing bacterial burden. Although randomized controlled trials on this topic are unlikely, large cohorts of prospectively collected data, particularly focusing on lower respiratory tract infections, may help clarify the optimal treatment approach.

## Supplementary Information

Below is the link to the electronic supplementary material.

Supplementary Material 1 (DOCX 22.4 KB)

## Data Availability

The datasets generated during and/or analysed during the current study are available from the corresponding author on reasonable request.
