# Supplementary material for: Is less always more? Ceftazidime/avibactam alone or in combination to treat infections caused by Pseudomonas aeruginosa: a retrospective observational multi-centre cohort study
Source: Eur J Clin Microbiol Infect Dis. 2026 Jul 14;45(10):3217–27. doi: 10.1007/s10096-026-05599-x (PMC13676522; doi:10.1007/s10096-026-05599-x)
Supplement: Supplementary file 1 [file 10096_2026_5599_MOESM1_ESM.docx]

**Supplementary materials**

**Is less always more? Ceftazidime/avibactam alone or in combination to treat infections caused by *Pseudomonas aeruginosa*: a retrospective observational multi-centre cohort study.**

**Summary**

[Clinical cure definitions according to type of infection	2](#_Toc227743921)

[Supplementary Table: Antibiotics given in combination with CZA	3](#_Toc227743922)

**Clinical cure definitions according to type of infection**

**Bloodstream infection:** patient alive and apyretic for the last 72 hours with improvement of vital signs and organ failures from baseline.

**Hospital-acquired pneumonia/ventilator-associated pneumonia (HAP/VAP):** patient alive and apyretic for the last 72 hours with reduction of respiratory support and/or improvement of PaO2 (or SpO2 when PaO2 is missing), with improvement or lack of progression of chest radiographic abnormalities.

**Complicated intra-abdominal infection (cIAI):** patient alive with complete resolution of physical signs or symptoms consistent with intra-abdominal infection (abdominal pain and/or tenderness, with or without rebound, localized or diffuse abdominal wall rigidity, abdominal mass, nausea/vomiting, and/or altered mental status) without further need of drainage or surgical intervention.

**Urinary tract infection/complicated urinary tract infection (UTI/cUTI):** patient alive and apyretic for the last 72 hours with resolution of frequency, urgency, dysuria, suprapubic pain, and/or flank pain.

**Acute bacterial skin structure and soft tissue infection (ABSSSI):** patient alive and apyretic for the last 72 hours with resolution or significant improvement of erythema, induration/swelling, suppuration.

# Supplementary Table: Antibiotics given in combination with CZA

|  | **CZA in**  **Combination therapy** |
| --- | --- |
|  | **N=70** |
| Antibiotic in combination treatment: |  |
| Meropenem | 5 (7.1) |
| Ciprofloxacin | 2 (2.9) |
| Amikacin | 19 (27.2) |
| Gentamicin | 3 (4.3) |
| Tobramycin | 2 (2.9) |
| Colistin | 15 (21.4) |
| Fosfomycin iv | 4 (5.7) |
| Aztreonam | 2 (2.9) |
| Meropenem + Colistin | 2 (2.9) |
| Ciprofloxacin + Colistin | 2 (2.9) |
| Ciprofloxacin + Amikacin | 1 (1,5) |
| Amikacin + Fosfomycin | 5 (7.1) |
| Amikacin + Colistin | 4 (5.7) |
| Gentamycin + Aztreonam | 1 (1,5) |
| Tobramycin + Fosfomycin | 1 (1,5) |
| Tobramycin + Colistin | 1 (1,5) |
| Results are expressed as counts and percentages. | |
